# A Self-Attention-Guided 3D Deep Residual Network With Big Transfer to Predict Local Failure in Brain Metastasis After Radiotherapy Using Multi-Channel MRI

**DOI:** 10.1109/JTEHM.2022.3219625

**Published:** 2022-11-04

**Authors:** Seyed Ali Jalalifar, Hany Soliman, Arjun Sahgal, Ali Sadeghi-Naini

**Affiliations:** Department of Electrical Engineering and Computer ScienceLassonde School of EngineeringYork University 7991 Toronto ON M3J 1P3 Canada; Physical Sciences PlatformSunnybrook Research Institute, Sunnybrook Health Sciences Centre Toronto ON M4N 3M5 Canada; Department of Radiation OncologyOdette Cancer CentreSunnybrook Health Sciences Centre 71545 Toronto ON M4N 3M5 Canada; Department of Radiation OncologyUniversity of Toronto 7938 Toronto ON M5T 1P5 Canada

**Keywords:** Attention mechanism, brain metastasis, deep learning, stereotactic radiotherapy, therapy outcome prediction

## Abstract

A noticeable proportion of larger brain metastases (BMs) are not locally controlled after stereotactic radiotherapy, and it may take months before local progression is apparent on standard follow-up imaging. This work proposes and investigates new explainable deep-learning models to predict the radiotherapy outcome for BM. A novel self-attention-guided 3D residual network is introduced for predicting the outcome of local failure (LF) after radiotherapy using the baseline treatment-planning MRI. The 3D self-attention modules facilitate capturing long-range intra/inter slice dependencies which are often overlooked by convolution layers. The proposed model was compared to a vanilla 3D residual network and 3D residual network with CBAM attention in terms of performance in outcome prediction. A training recipe was adapted for the outcome prediction models during pretraining and training the down-stream task based on the recently proposed big transfer principles. A novel 3D visualization module was coupled with the model to demonstrate the impact of various intra/peri-lesion regions on volumetric multi-channel MRI upon the network’s prediction. The proposed self-attention-guided 3D residual network outperforms the vanilla residual network and the residual network with CBAM attention in accuracy, F1-score, and AUC. The visualization results show the importance of peri-lesional characteristics on treatment-planning MRI in predicting local outcome after radiotherapy. This study demonstrates the potential of self-attention-guided deep-learning features derived from volumetric MRI in radiotherapy outcome prediction for BM. The insights obtained via the developed visualization module for individual lesions can possibly be applied during radiotherapy planning to decrease the chance of LF.

## I. Introduction

About 20% of patients with extracranial malignancies develop brain metastases (BM) [1]. It is estimated that between 70,000 to 400,000 new cases of BM are diagnosed each year in the United States [2]. Because of increasing access to neuroimaging and developments in systemic therapies for patients with metastatic disease, as well as increased physician and patient awareness of BMs, the incidence of BM is expected to increase among cancer patients [1].

The survival of patients with BM depends on timely diagnosis and effective therapy. The major therapeutic options for metastatic brain tumours include surgery, radiation therapy, and/or chemotherapy. Surgical resection is recommended for patient with large solitary tumours in an accessible location [3]. The three principal modalities of radiation therapy (RT) for BM are whole-brain radiation therapy (WBRT), single-fraction stereotactic radiosurgery (SRS), and hypo-fractionated stereotactic radiotherapy (SRT). While WBRT has been the main treatment for patients with multiple BM [4], there has been a move away from WBRT to SRT and SRS due to adverse side-effects associated with WBRT such as fatigue and cognitive deterioration [5], [6].

Because of various tumour and/or patient-related characteristics such as tumour size, location, and histology as well as the patient’s genetics, age and performance status, local response of BM tumours to radiation varies among patients. This is true even when standardized dose/fraction regimens are administrated [7]. The local response to RT is classified as either local control (LC; stable or shrinking tumour that is indicative of a stable disease, partial response, or complete response,) or local failure (LF; enlarging tumour associated with a progressive disease) based on tumour size changes on follow-up structural serial imaging [8]. However, it could take months for a local response to be visible on follow-up scans. Given that the median survival of BM patients following RT can be between 5 months and 4 years [9], [10], early detection of LF after RT potentially permit effective adjustments in treatment that lead to enhanced therapy outcomes, patients’ survival, and their quality of life.

Following the successful application of artificial intelligence (AI) methods in diagnostic imaging [11], [12], AI-based cancer imaging analysis is now being used to meet other and more complex clinical challenges [13], [14]. These methodologies have the capacity to uncover previously unknown features from routinely acquired medical images. Quantitative and semi-quantitative features, which are often beyond human perception, can be derived from obtained neuroimaging data. These features can potentially be applied to develop machine learning models to address crucial clinical challenges such as therapy outcome assessment or treatment response prediction. Radiomics is a relatively new transformational research domain that adapts high-throughput approaches for mining of large-scale medical imaging datasets to identify quantitative features (biomarkers) for different diagnostic and prognostic applications [15]. Multiple studies have shown links between radiomic signatures of tumours and their phenotypic, genomic, and proteomic profiles [16]. Several studies have also demonstrated the efficacy of radiomic-based machine learning models in therapy outcome prediction [17], [18], including local response of BM to radiotherapy [19], [20], [21].

Compared to hand-crafted radiomic features, the application of deep learning in medical imaging could possibly address more complicated challenges, particularly when large relevant datasets are available. Deep learning models have shown great promise in recognizing important and distinctive aspects of medical image data in various applications including cancer therapeutics [22], [23], [24]. Deep models, and especially convolutional neural networks (CNNs), can detect complex textural patterns in tissue, distinguish between malignant and benign cells, and possibly derive information from tumour images for therapy outcome prediction [25], [26], [27]. Accordingly, the CNNs can potentially outperform the traditional radiomic models in diagnostic and prognostic applications for precision oncology by detecting patterns in medical images that are not captured by closed-form mathematical definitions of hand-crafted radiomic features [28], [29], [30]. A recent publication from our group shows that the deep-learning features derived from 2D MRI slices outperform the standard clinical variables in predicting radiotherapy outcome in BM [31].

Attention mechanisms in deep learning were introduced in the field of computer vision with the goal of imitating the human visual system’s ability to naturally and effectively discover prominent regions in complex scenes [32]. An attention mechanism in a vision system can be thought of as a dynamic selection process that is implemented by adaptively weighing features based on the relevance of the input. Over the past few years, attention mechanism has played an increasingly important role in different computer tasks, including image classification [33], object detection [34], semantic segmentation [35], and 3D vision [36]. The attention mechanism has shown promise in medical imaging analysis, especially when the problem is not as straightforward as generic image classification, where the well-defined object of interest is usually in the image center [37]. A number of previous studies have applied attention mechanisms to provide more powerful architectures capable of catching subtle features covered in medical images. Guan et al. [38] proposed a three-branch attention guided convolution neural network (AG-CNN) which learns from disease-specific regions through a local branch to reduce noise and improve alignment, with a global branch to compensate for the lost discriminative cues by the local branch. Using a fusion branch to combine the local and global cues, their model could achieve a new state-of-the-art performance in classifying images of the ChestX-ray14 dataset [38]. Rao et al. [39] conducted an experimental research to investigate the contribution of various attention mechanisms including squeeze-and-excitation (SE) [33], global context (CG) [40], and convolutional block attention module (CBAM) [41] to the performance of deep classification models for different imaging modalities including x-ray, MRI, and CT. The experimental results show that the attention mechanisms enable standard CNN models to focus more on semantically important and relevant content within features, with improved area under the receiver operating characteristic (ROC) curve (AUC) for all classification models investigated [39]. Furthermore, the CBAM outperformed the other two attention mechanisms in several experiments on different imaging datasets. Shaik et al. [42] proposed a multi-level attention mechanism for the task of brain tumour classification. The proposed multi-level attention network (MANet) combines spatial and cross-channel attention, focusing on tumour region prioritization while also preserving cross-channel temporal connections found in the Xception backbone’s semantic feature sequence [42]. They benchmarked their framework on BraTS [43] and Figshare [44] datasets where their model outperformed several models proposed previously for the brain tumour classification task [42].

This translational study introduces an innovative transformer-convolutional deep learning model for predicting the LC/LF outcome in BM treated with SRT using two-channel MRI acquired at pre-treatment. A novel attention-guided 3D residual network architecture has been developed with embedded self-attention modules [45], [46], [47] and compared with another residual network with 3D CBAM as the attention mechanism. A training recipe has been adapted for the therapy outcome prediction models during pretraining and training the down-stream task based on the recently-proposed big transfer (BiT) principles [48]. A new 3D visualization method has been introduced to illustrate the impact of different regions throughout the lesion volume upon the network’s prediction of the therapy outcome. The results demonstrate that incorporating the attention mechanisms into the vanilla 3D residual network improves its performance in outcome prediction considerably, with the self-attention mechanism outperforming the CBAM in terms of accuracy, AUC, and F1-score. Further, the adapted BiT-based recipe for pretraining and hyperparameter tuning improves the deep models’ performance in therapy outcome prediction.

## II. Methods and Procedures

### A. Data Acquisition

This study was carried out in compliance with the institutional research ethics board approval from Sunnybrook Health Sciences Centre (SHSC), Toronto, Canada. Data were obtained from 124 BM patients treated with hypo-fractionated SRT (5 fractions). In this study, the baseline treatment-planning MRI including contrast-enhanced T1-weighted (T1w), and T2-weighted-fluid-attenuation-inversion-recovery (T2-FLAIR) images were applied for therapy outcome prediction. The MRI scans were acquired using a 1.5 T Ingenia system (Philips Healthcare, Best, Netherlands) and a 1.5 T Signa HDxt system (GE Healthcare, Milwaukee, WI, USA). The T1w and T2-FLAIR images had an in-plane image resolution of 0.5 mm and a slice thickness of 1.5 mm and 5 mm, respectively. The treatment-planning tumour contours delineated by expert oncologists as well as the edema contours outlined under their supervision were also included in the dataset. The dataset (124 patients with 156 lesions) was randomly partitioned at patient level into a training set (99 patients with 116 lesions) that was used for model development and optimization, and an unseen test set (25 patients with 40 lesions) that was applied for independent evaluation of the models. From the training set, 10 patients with 15 lesions were randomly selected as the validation set for optimizing the model hyperparameters.

The patients were scanned with MRI after SRT on a two to three-month follow-up schedule. A radiation oncologist and a neuroradiologist determined the local response for each lesion separately after monitoring it on serial MRI using the RANO-BM [8] criteria. The outcome (LC or LF) was determined for each lesion in the last patient follow-up. Serial imaging (including perfusion MRI) and/or histological confirmation were used to diagnose adverse radiation effect (ARE) and distinguish it from progressive disease [49], in accordance with the report by Sneed et al. [50]. Following these criteria, a total of 93 lesions were categorized as LC while 63 lesions were labeled as LF.

### B. Preprocessing

All MR images were resampled to a size of 512 $\times $ 512 $\times $ 174 voxels (voxel size: 0.5 $\times $ 0.5 $\times $ 1 mm^3^). An affine registration method was used to co-register the T1w and T2-FLAIR images. Skull stripping was performed on all MR images. The voxel intensities in each skull-stripped MRI volume were normalized between 0 and 1. To ensure a lesion-level local outcome prediction the size of the smallest sub-volume enclosing the tumour and edema (lesion) and their 5-mm outer margin [51], was identified for all lesions. A sub-volume of 128 $\times $ 128 $\times $ 83 voxels was determined as a fit standard to encompass the entire region of interest (ROI) described above for all individual lesions. The standardized sub-volumes were then cropped from the T1w and T2-FLAIR images and concatenated for each lesion as two channels of data, generating the input to the neural networks with a size of 128 $\times $ 128 $\times $ 83 $\times2$ voxels. The ROI masks (tumour +5-mm margin for T1w; tumour + edema +5-mm margin for T2-FLAIR) were generated using the tumour and edema contours and applied to mask out the areas outside the ROI for each lesion.

### C. Network Overview

**FIGURE 1.**
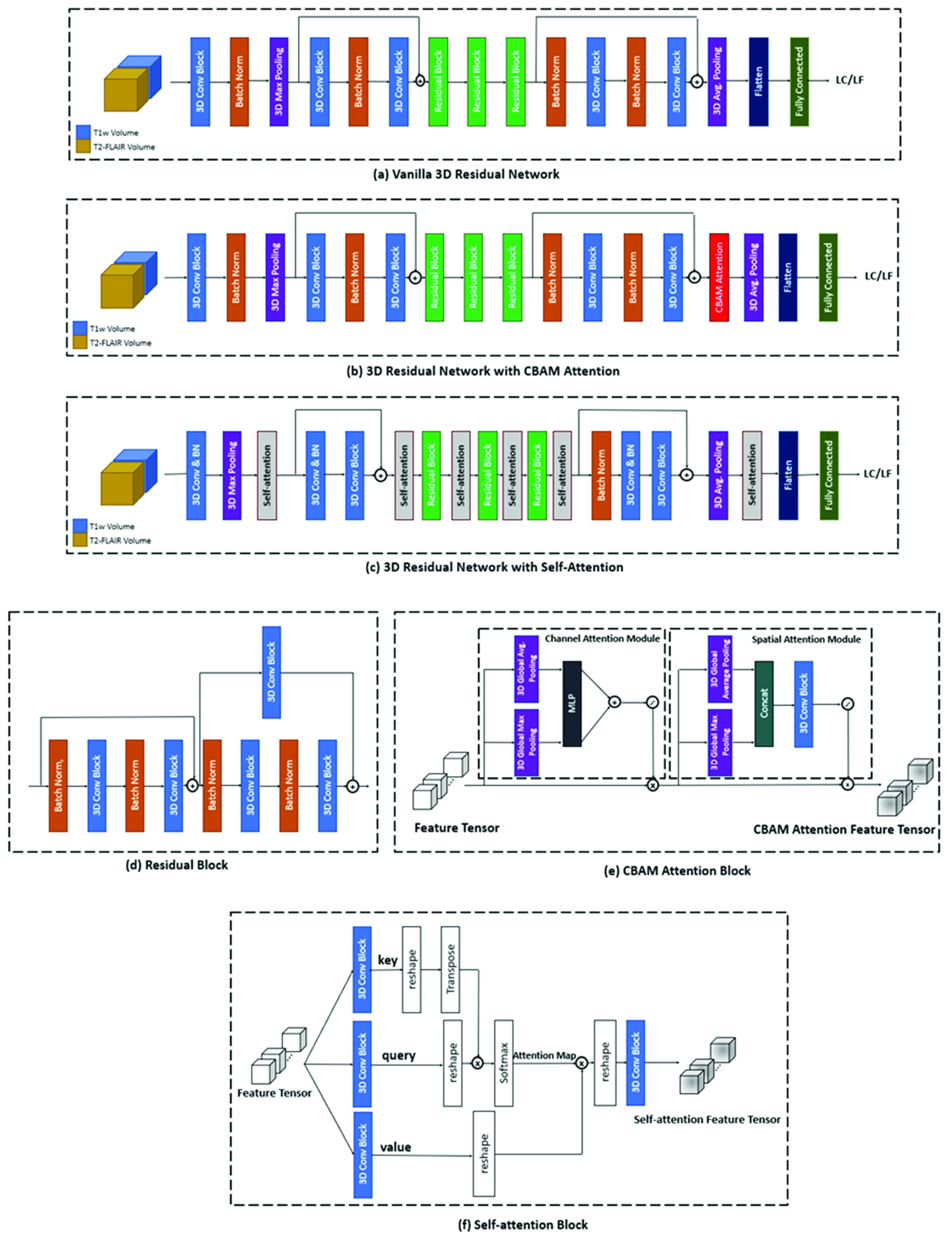
Architecture of (a) vanilla 3D residual network (baseline), (b) the 3D residual network with CBAM attention, (c) the proposed self-attention-guided 3D residual network, (d) the residual block in 3D residual network, consisting of residual connections, (e) the CBAM attention block consisting of the channel and spatial attention modules, and (f) the self-attention block consisting of the key, query, and value tensors that generates the final self-attention feature tensor.

The backbone of the proposed network architecture is a vanilla 3D extension of deep residual networks (ResNets), first introduced by He et al. [52], [53] Instead of learning unreferenced functions, ResNets learn residual functions with reference to the layer inputs. Also, rather than expecting each few stacked layers directly fit a desired underlying mapping, ResNets let these layers fit a residual mapping. Formally, instead of directly mapping the desired underlying function $H(x)$, the stacked nonlinear layers fit another mapping of $F\left ({x }\right):=H\left ({x }\right)-x$. This way, the original mapping recast into $F\left ({x }\right)+x$. Residual connections allow for increased depth while addressing the vanishing gradient problem and are also easier to optimize [52]. Our vanilla 3D residual network (Figure 1.a) is inspired by the architecture of ResNet-18, but instead of 2D convolution layers, our network employs 3D convolution with kernel size of 7 $\times $ 7 $\times7$ and 3D pooling layers to handle the 3D nature of MRI volume.

To improve the performance of our 3D residual network in processing multi-channel MRI volumes, we explored incorporating two different attention mechanisms into the architecture, CBAM, and self-attention [46]. CBAM is a simple yet effective attention module which infers attention maps along two separate dimensions (channel and spatial) sequentially [41]. The attention maps are then multiplied by the feature tensors to produce the refined feature tensors. Formally, CBAM has two sequential submodules and, given an input feature tensor $x\in R^{X\times Y\times Z\times C}$, it sequentially infers 1D channel attention vector $M_{c}\epsilon R^{C}$ and a 3D spatial attention map $M_{s}\epsilon R^{X\times Y\times Z}$. The developed 3D residual network with CBAM attention is depicted in Figure 1.b. The CBAM attention module (Figure 1.e) is embedded right before the average pooling and fully-connected layers to refine features before classification. The refined features are then flattened and fed to the fully-connected layer for classification.

Additionally, we introduced a novel transformer-convolutional network architecture by incorporating self-attention modules into the 3D residual network (Figure 1.c). The convolution operator in CNNs only conducts local operations and has a local receptive field, but the self-attention mechanism can perform non-local operations and capture long-range dependencies and global information within the input images [54]. The self-attention method is based on the covariance between the elements of feature tensors [55]. Formally, a self-attention function can be described through mapping the input feature tensor to a query, a key, and a value tensor. The tensor mappings are performed using 3D 1 $\times $ 1 $\times1$ convolutions. Each element of the output self-attention feature tensor is a linear weighted sum of the elements of the value tensor. The query tensor defines which “values” to focus on for the learning process, while the key and value tensors carry the transformed features extracted from MRI volume. Given that key is $k\left ({x }\right)=W_{k}x$, query is $q\left ({x }\right)=W_{q}x$, and value is $v\left ({x }\right)=W_{v}x$ where $W_{k}$, $W_{q}$, $W_{v}$ are learnable weights of the $1\times 1\times1$ convolution filters and $x$ is the feature tensor from the previous layer, the self-attention map $\alpha $ could be calculated as $\alpha _{i,j}=\frac {\mathrm {exp}(q\left ({x_{i} }\right)k\left ({x_{j} }\right)^{T})}{\sum \nolimits _{i=1}^{n} {\mathrm {exp}(q\left ({x_{i} }\right)k\left ({x_{j} }\right)^{T})}}$. Figure 1.f shows the architecture of the 3D self-attention block incorporated into the proposed 3D residual network with self-attention. For performing the matrix multiplications in this block, the query, key, and value tensors ( $\in R^{X\times Y\times Z\times C}$) are reshaped into matrices ( $\in R^{XYZ\times C}$) and, at the end, reshaped back into tensors of the initial size. The final 1 $\times $ 1 $\times1$ convolution block ensures that the number of channels of the input and output feature tensors stays the same. The 3D self-attention module facilitates capturing long-range inter/intra slice dependencies, hence is added to the architecture after each residual block to ensure deriving such dependencies along with the convolution layers that mostly capture local features and dependencies. More details on the network architectures have been provided in the Supplementary Materials.

### D. Big Transfer and Training Details

Transfer of pretrained models on the target task improves sample efficiency and simplifies hyperparameter tuning when training deep neural networks [48]. Inspired by the work of Kolesnikov et al. [48], we followed the subsequent scheme for pretraining/training the outcome prediction models:

1. The network was first pretrained on the UCF101 dataset [56] for the task of activity recognition and subsequently on the BraTS dataset [43], [57], [58] for the task of classifying brain tumour types using MRI.
2. During pretraining, all batch normalization [59] layers were replaced with group normalization [60] and weight standardization [61] was used in all convolutional layers. The combination of group normalization and weight standardization with large batches has a significant impact on transfer learning [62]. Also, due to the requirement to update running statistics, batch normalization is detrimental for the transfer [48].
3. During fine-tuning on the main dataset, we used BiT-HyperRule, a heuristic method for hyperparameter selection based on image resolution and number of datapoints as presented by [48]. The models were trained using the stochastic gradient descent (SGD) optimization algorithm with an initial learning rate of 0.003, momentum of 0.9, batch size of 4, and an early stopping based on the validation loss. Data augmentation was performed using horizontal flipping. During fine-tuning, the learning rate was decayed by a factor of 10 at 40%, 60% and 80% of the training steps.

All experiments were performed in Python. The models were developed and evaluated using Keras [63] with TensorFlow [64] backend. The performance metrices were calculated using scikit-learn package [65]. the matplotlib library was used [66] for visualization. The models were trained using four GeForce RTX TI 2080 graphic cards. The training process took 5 hours ( $\sim 33\text{M}$ parameters), 6 hours ( $\sim 33\text{M}$ parameters) and 10 hours ( $\sim 42\text{M}$ parameters) for 3D residual network, 3D residual network + CBAM attention, and 3D residual network + self-attention respectively. The total inference time for a single input is 6ms, 7ms, and 12ms for 3D residual network, 3D residual network + CBAM attention, and 3D residual network + self-attention, respectively.

### E. Visualization of Network Decision Basis

A new 3D visualization algorithm was implemented to accompany the outcome prediction framework and show how different areas within the volumetric region of interest on the input images contribute to the prediction of network for each lesion. The visualization module provides a 3D heatmap color-coding the relevance of distinct peri-/intra-lesion areas on multi-channel volumetric MRI to the decision of network and may be applied to analyze the reasoning behind the predicted outcome for each case. The applied visualization method combined a modified version of the prediction difference analysis (PDA) with a sliding window analysis approach [67]. A 2 $\times $ 2 $\times1$ voxel sliding black cube (1 $\times $ 1 $\times $ 1 mm) was iteratively applied to block a tiny area of the input image. The occluded input was fed to the trained network to predict the associated therapy outcome. In each iteration, the absolute difference in the network’s output probability (i.e., $\vert p_{input}-p_{occluded\_{}input}\vert$) was calculated and applied as a measure of contribution of the occluded cube to generate the volumetric heatmap. This method generates a point cloud where each point in the cloud maps to a region within the MRI volume. For 3D visualization of generated heatmap, the heatmap voxels were considered as a point cloud with each point maps to a region within the MRI volume. A surface reconstruction technique was adapted to create a 3D heatmap out of the point cloud on any desired surface within the volumetric ROI. Specifically, the cloud points located on the ROI surface were identified and the normal orientation of the point cloud was calculated at each surface point using a minimum spanning tree with the number of neighbours set to 3 for building the tree [68]. The estimated normal orientations were applied in conjunction with, the Poisson reconstruction technique [69] to build a smooth surface mesh from the point cloud. The Poisson surface reconstruction technique creates a 3D mesh from a dense point cloud by reducing the difference between the surface normal directions of the reconstructed surface and the 3D points in the point cloud [70]. The proposed 3D visualization framework can assist clinicians to get insight into how the network has reached its decision and help to validate the network’s decisions by generating meaningful heatmaps. More details on the visualization framework have been provided in Supplementary Materials.

## III. Results

The patients (average age: 62 ± 15 years; 40% male and 60% female) had an average tumour size of 2 ± 1.03 cm and an average GPA of 2.2. The demographic and clinical attributes of the patients in this study are presented in Supplementary Table S1.

**FIGURE 2.**
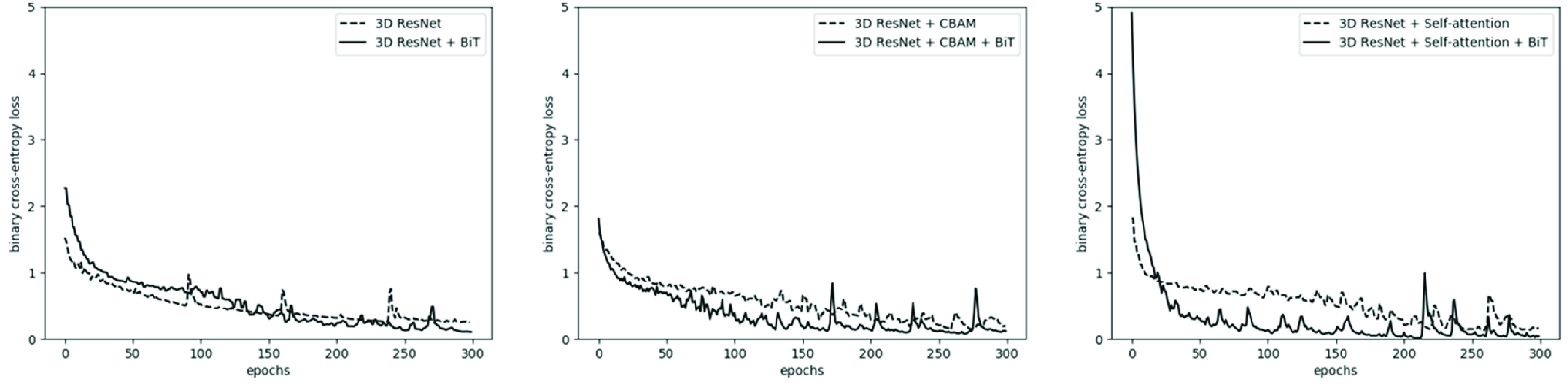
Training loss of the three models investigated in this study before and after applying BiT training scheme. Following the BiT training recipe generally led to faster convergence, smaller loss, and better performance overall.

**FIGURE 3.**
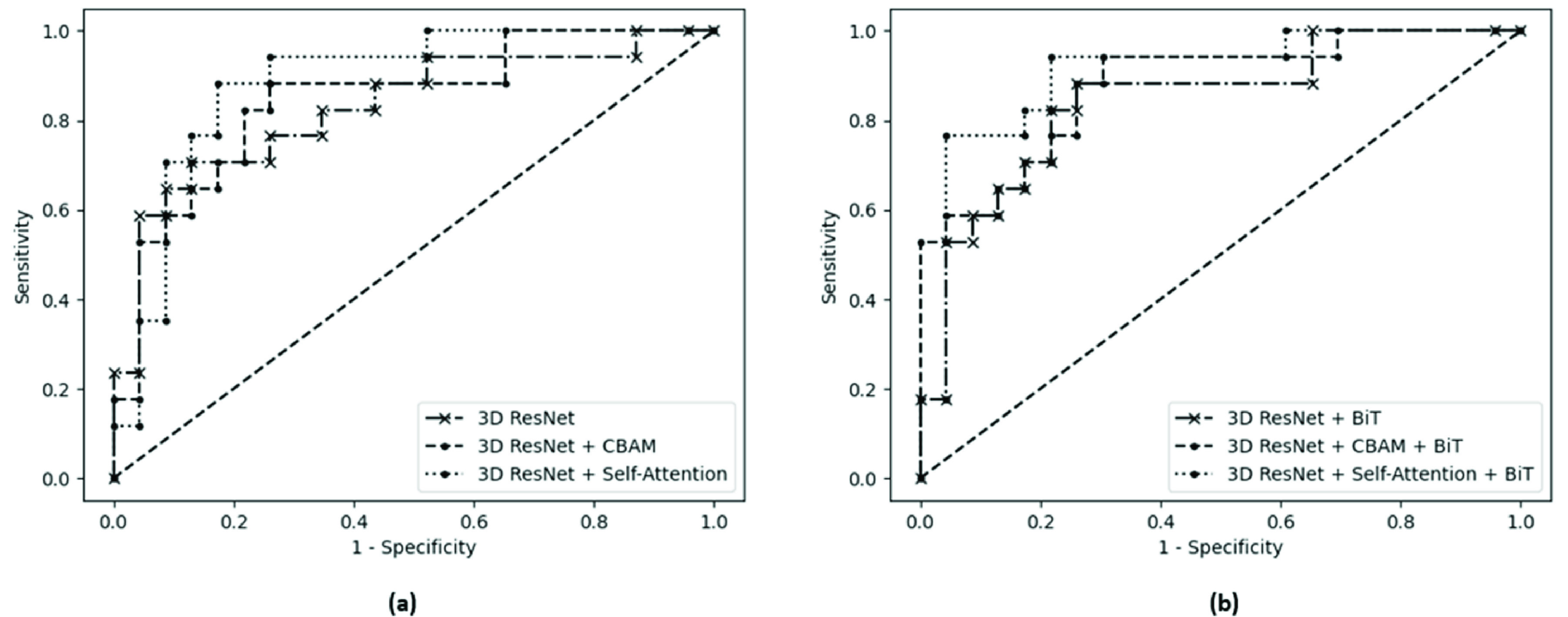
The ROC curves for (a) vanilla 3D residual network, 3D residual network + CBAM, and 3D residual network + self-attention, and (b) the same models trained with the BiT scheme.

Figure 2 shows the training loss over 300 epochs for the models in this study before and after applying the BiT training scheme. The vanilla 3D residual network pretrained on the UFC101 and BraTS datasets (without BiT scheme) is the baseline model of this study. In pretraining/training of the models without the BiT recipe, the batch normalization layers were not replaced with group normalization and the weight standardization and BiT-HyperRule were not applied. Table 1 presents the performance of different models investigated in this study for radiotherapy outcome prediction. A careful investigation of Figure 2 and Table 1 demonstrates that incorporating the BiT scheme in development of the deep models for outcome prediction generally improves their performance in terms of convergence, loss, F1-score, and AUC on the independent test set. The F1-score and AUC may be considered the most important metrics presented in Table 1 because of the imbalance exists in the dataset. Specifically, following the BiT training scheme, the models improved their AUC on the test set from 0.83 to 0.84, 0.87 to 0.88, and 0.88 to 0.91 for the 3D residual network, 3D residual network + CBAM attention, and 3D residual network + self-attention, respectively. From a different perspective, incorporating attention mechanisms also improved the model performances in terms of accuracy, AUC, and F1-score. While the vanilla 3D residual network could achieve an F1-score of 75% on the test set, the 3D residual network + CBAM attention improved the F1-score by 2.8%. The F1-score was improved by 3.8% compared to the baseline model by including the self-attention mechanism in the 3D residual network. In particular, the proposed transformer-convolutional network architecture with BiT training demonstrated the best performance in terms of accuracy, AUC, and F1-score, with 8% and 5% improvements in AUC and F1-score, respectively, compared to the baseline model, on the independent test set. This is a considerable improvement in model’s performance given the complicated task at hand. Further, the proposed model resulted in the most balanced sensitivity and specificity values compared to the other models despite the imbalanced dataset applied in the study. Figure 3 shows the ROC curves for different models investigated in this study.

**TABLE 1:** Results of Radiotherapy Outcome Prediction for Different Models. Acc: Accuracy; Sens: Sensitivity; Spec: Specificity

Network	Validation Set					Independent Test Set				
	Acc.	Sens.	Spec.	AUC	F1-Score	Acc.	Sens.	Spec.	AUC	F1-Score
3D Residual Network	80%	66.7%	88.9%	0.84	72.7%	80%	71%	87%	0.83	75%
3D Residual Network + BiT	80%	83.3%	77.8%	0.86	76.9%	80%	82.4%	78.%	0.84	77.8%
3D Residual Network + CBAM Attention	80%	83.3%	77.8%	0.88	76.9%	80%	82.4%	78.2%	0.87	77.8%
3D Residual Network + CBAM Attention + BiT	80%	100%	66.7%	0.88	80%	80%	88.2%	73.9%	0.88	78.9%
3D Residual Network + Self-attention	86.7%	83.3%	88.9%	0.89	83.3%	82.5%	76.5%	87%	0.88	78.8%
3D Residual Network + Self-attention + BiT	86.7%	83.3%	88.9%	0.93	83.3%	82.5%	82.4%	82.6%	0.91	80%

**FIGURE 4.**
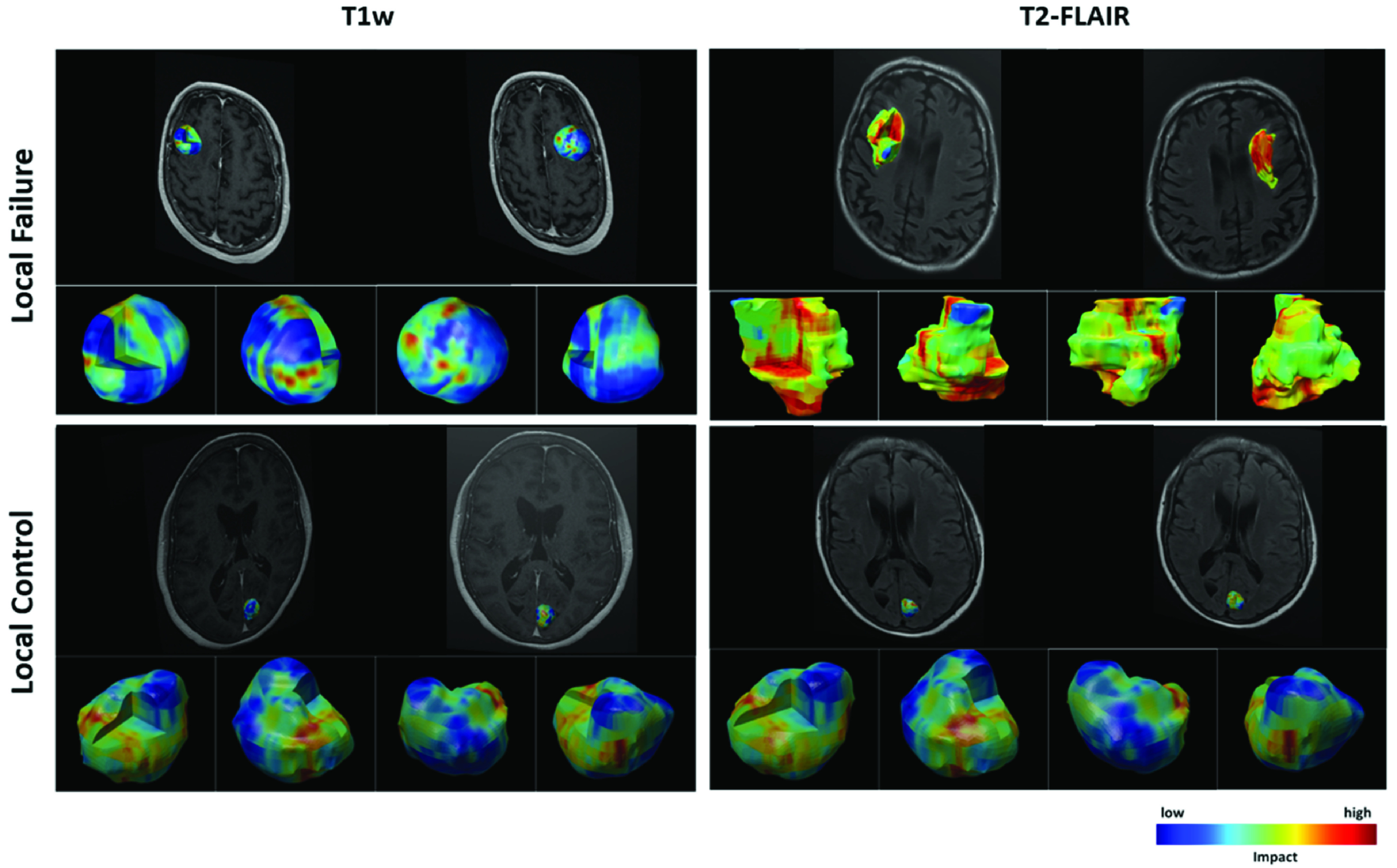
3D visualization heatmaps corresponding to the two input channels (T1w and T2-FLAIR) of the 3D residual network with self-attention and BiT training for two representative lesions, one with an LF (top) and the other one with an LC (bottom) outcome. The user can inspect any desired area on the lesion/margin surface or inside the volumetric ROI and their correspondence with the MRI channels.

An explanation of how an attention mechanism helps improving the performance of the deep models in therapy outcome prediction is as follows: characteristics of different regions within tumour and peritumoral areas on MRI contribute unequally to the likelihood of local response. Several studies demonstrate that tumour margin areas on MRI carry invaluable information regarding the responsiveness of BM to radiotherapy with possibly higher importance for prediction modeling compared to the core areas [51], [71]. Attention mechanisms help the network to capture the subtle information latent within MRI by filtering out irrelevant data and focusing on regions which truly contribute to the network decisions. Moreover, self-attention facilitates capturing long-range dependencies inside the MRI volume, an important concept that simple 3D convolutional layers are not capable of because of their local nature and limited field of view. Comparing the performance of 3D residual network + CBAM attention and the vanilla 3D residual network, the former has outperformed the latter in terms of AUC and F1-Score, although the number of parameters is almost the same for these networks. This shows the benefit of incorporating attention mechanisms in this setting while it may not increase the network complexity considerably. Our further experiments with the 3D residual network with more parameters ( $\sim 40\text{M}$ parameters) when extra layers were added to the network resulted in overfitting. This implies that the 3D residual network + self-attention does not simply benefit from the increased number of network parameters but mainly from the structure of the attention layers incorporated. Figure 4 demonstrates the 3D visualization heatmaps for two representative lesions generated using the technique introduced in Section II-E. The heatmaps show the contribution level of different regions within the volumetric ROI on the prediction of the proposed attention-guided model for each lesion in terms of local outcome. The 3D heatmaps can aid clinicians to examine the lesion volume thoroughly and inspect impactful regions for a predicted outcome which can eventually support their decision making in assessment, diagnosis, and treatment planning.

## IV. Discussion and Conclusion

An end-to-end 3D convolutional deep learning architecture with self-attention was introduced in this study to predict the local outcome in BM after radiotherapy. By employing 3D residual blocks in the proposed model, we investigated the possibility of early prediction of LF in BM treated with SRT using T1w and T2-FLAIR MRI volumes acquired at baseline. We further investigated the effect of incorporating attention mechanisms into the 3D residual network. The results show that the proposed model with self-attention mechanism outperforms the vanilla 3D residual network and the 3D residual network with CBAM attention in terms of accuracy, AUC, and F1-score. The proposed architecture combines residual learning with self-attention mechanism, allowing for full utilization of both global and local information while avoiding information loss. Specifically, the self-attention mechanism in the model takes into account long-range dependencies in the input MRI volumes while the residual connections allow the extracted information to persist throughout the network. We further improved the model’s performance by following the BiT scheme for pretraining and hyperparameter tuning. A 3D visualization module was developed and coupled with the framework to show the important areas of lesion on MRI with higher impact on the model’s decision. The visualization results confirm the findings of previous studies that the characteristics of tumour/lesion margin areas on T1w and T2-FLAIR images are important for predicting local outcome in BM treated with radiation therapy. In particular, these regions are among the high-impact regions to the predictions made by the proposed deep learning model with more attention gained from the model for therapy outcome prediction.

The findings of this study demonstrate the feasibility of early prediction of radiotherapy outcome for BM using only the features extracted from multi-modal MRI volumes. This study highlights the effect of adding attention mechanism to deep networks and the importance of pretraining in transferring knowledge to the fine-tuning step. When dealing with large models and large datasets (which is usually the case during pretraining) adhering to the BiT recipe allows for optimized training during the up-stream task and a computationally inexpensive fine-tuning protocol during the down-stream task to avoid a complex and costly hyper-parameter search. The obtained results are promising and encourage future studies on larger patient populations. The results of this study were obtained using an independent test set that was kept unseen during the model training and optimization. However, for a more rigorous evaluation of the efficacy and robustness of the models in the clinic, further investigations should be performed on larger patient cohorts and preferably with multi-institutional data.

## Supplementary Materials

Supplementary materials
