# Supplementary material for: A Self-Attention-Guided 3D Deep Residual Network With Big Transfer to Predict Local Failure in Brain Metastasis After Radiotherapy Using Multi-Channel MRI
Source: IEEE J Transl Eng Health Med. 2022 Nov 4;11:13–22. doi: 10.1109/JTEHM.2022.3219625 (PMC9721353; doi:10.1109/JTEHM.2022.3219625)
Supplement: Supplementary materials [file supp1-3219625.pdf]

Supplementary Materials

A Self-Attention-Guided 3D Deep Residual Network with Big Transfer to Predict Local Failure in Brain Metastasis after Radiotherapy using Multi-Channel MRI

Seyed Ali Jalalifar[(1)], Hany Soliman[(2,3,4)], Arjun Sahgal[(2,3,4)], and Ali Sadeghi-Naini[(1,2,4)]

(1) Department of Electrical Engineering and Computer Science, Lassonde School of Engineering, York University, Toronto, ON, Canada

(2) Department of Radiation Oncology, Odette Cancer Centre, Sunnybrook Health Sciences Centre, Toronto, ON, Canada

(3) Department of Radiation Oncology, University of Toronto, Toronto, ON, Canada

(4) Physical Sciences Platform, Sunnybrook Research Institute, Sunnybrook Health Sciences Centre, Toronto, ON, Canada

Details of the Deep Network Architectures

3D Residual Network

The 3D residual network applied in this study is an extension of residual networks or ResNets with 3D components instead of 2D ones. Historically, the trend of making networks deeper to increase their modeling capabilities was hindered by vanishing gradient. After numerous applications of the chain rule, the gradients from which the loss function is derived simply drop to zero when the network is too deep. As a consequence, the weights at higher layers never update their values, hence no learning takes place. By introducing skip connections, gradients can flow backward from deeper layers to initial filters directly via these connections. Skip connections enable the network to easily model the identity function, where the output of a function becomes its input. More specifically, instead of learning the output function $H(x) = f(x)$, the output is changed into $H(x) = f(x) + x$. Simply, by setting $f(x) = 0$, $H(x)$ becomes the identity function. Figure 1.a shows the architecture of the 3D residual network applied in this study. The most important part of the architecture is the stack of residual blocks and skip connections to preserve the gradient. Further to preserving gradient, another reason that skip connections prove useful is the fact that the learned features correlate to lower semantic information retrieved from the input in prior levels. That information become too abstract if the skip connections are not utilized in this architecture.

CBAM: Convolutional Block Attention Module

When it comes to feed-forward convolutional neural networks, CBAM is a simple yet effective attention module. Given an intermediate feature map, the module progressively infers attention maps along two different dimensions, *i.e.*, channel and spatial, and then multiplies the attention maps by the input feature tensors to perform adaptive feature refinement on the intermediate feature tensors. The fact that CBAM is a lightweight and universal module means that it can be smoothly integrated into any CNN architecture with minimal overhead and that it is trainable from start to finish alongside the base CNNs.

As mentioned above, CBAM consists of two sequential separate attention mechanisms, channel attention, and spatial attention. Because each channel of a feature tensor may be considered as a feature detector, channel attention is focused on 'what' is significant in the context of an input image when using feature tensors. Channel attention begins by aggregating spatial information from the feature tensor using both average-pooling and max-pooling processes, resulting in the generation of two separate spatial context descriptors for each feature map: F^c_{avg}

and F_{max}^c, which denote average-pooled features and max-pooled features, respectively. Afterwards, both descriptors are forwarded to a shared network, which generates the channel attention map $M_c \in R^{1\times1\times1\times C}$, where C is the number of channels. The shared network is made up of a multi-layer perceptron (MLP) with one hidden layer. Following the application of the shared network to each descriptor, the resulting feature tensors are combined by applying element-wise summing to form a single feature tensor. To summarize, the channel attention module is computed as $M_c(F) = \sigma(MLP(AvgPool(F)) + MLP(MaxPool(F)))$, where σ denotes the sigmoid function.

The next step is to compute spatial attention. In order to build a spatial attention map, the spatial attention module uses the inter-spatial relationship between features. At the other end of the spectrum from channel attention, spatial attention focuses on 'where' informative features are located in the image, and it is considered a complement to the channel attention. Using average-pooling and max-pooling operations along the channel axis, the spatial attention map can be calculated. A convolution layer is applied to the concatenated feature descriptor in order to construct a spatial attention map based on it, *i.e.*, $M_s(F) \in R^{X\times Y\times Z}$. The channel information in the feature tensor is aggregated via the use of two pooling processes, resulting in the generation of two 3D maps: $F_{avg}^s \in R^{X\times Y\times Z\times 1}$ and $F_{max}^s \in R^{X\times Y\times Z\times 1}$, that denote the average-pooled features over the channel, and the max-pooled features, respectively. Formally, $M_s(F) = \sigma(f^{7\times7\times7}([F_{avg}^s; F_{max}^s]))$, where σ is the sigmoid function and $f^{7\times7\times7}$ is the convolution function with the filter size of $7 \times 7 \times 7$.

The channel and spatial modules are applied to the intermediate feature maps sequentially and output the refined features. Figure 1.e shows the architecture of CBAM. In our proposed architecture, the CBAM block was added right before the 3D average pooling layer to filter out irrelevant information and focus on important details for classification. Figure 1.b shows the proposed architecture augmented with CBAM.

The Self-attention Module

A self-attention module is defined as a tensor mapping that transforms the input tensor to a query, a key, and a value tensor. The key and value are learned features extracted by convolution blocks, and the query determines which values to focus on for the learning process. The role of 3D convolution blocks ($1 \times 1 \times 1$ convolutions) before the key, query, and value is to perform linear transformations on the input feature tensors. The key, query, and value vectors are denoted by

$k(x)$, $q(x)$, and $v(x)$ and are calculated as $k(x) = W_k x$, $q(x) = W_q x$, and $v(x) = W_v x$, where W_k, W_q, and W_v are all $1 \times 1 \times 1$ convolution filters and x is the feature tensor coming from the previous layer. After reshaping to permit matrix multiplications, $k(x), q(x)$ and $v(x) \in R^{N \times C}$, where C is the number of channels and $N = X \times Y \times Z$ is the number of elements in the input feature tensor. The self-attention map α can be calculated as $\alpha_{i,j} = \frac{\exp\left(q(x_i)k(x_j)^T\right)}{\sum_{i=1}^{n} \exp\left(q(x_i)k(x_j)^T\right)}$. $\alpha_{i,j}$ is the correlation between the feature element i and other feature elements, and j is the index of corresponding output position. The output of the attention branch is $o = (o_1, o_2, \ldots, o_N)^T \in R^{N \times C}$, where $o_j = \sum_{i=1}^{N} \alpha_{i,j} v(x_i)$. Finally, a $1 \times 1 \times 1$ convolution (W_o) is applied to the reshaped output ($\in \mathbb{R}^{X \times Y \times Z \times C}$) to keep the number of channels consistent between the input and output feature tensors of the attention layer ($Output = W_o o$). Figure 1.f shows the self-attention block. Our proposed self-attention-guided 3D residual network architecture is demonstrated in Figure 1.c. The 3D self-attention module is added to the architecture after each residual block to ensure deriving long-range dependencies along with the convolution layers that mostly capture local features and dependencies.

3D Visualization

In order to provide explainability to the model, we proposed a framework for creating 3D heatmaps of the importance that highlight areas with the most contribution to the network's decision. For each voxel (or cubic super-voxel) in the MRI volume (or volumetric ROI within MRI), the importance is calculated by the absolute difference in network's output probability with and without that specific voxel. More specifically, the impact is defined as $impact = |p(x) - p(x_{/i})|$, were $p(x_{/i})$ is the output probability of the network after occluding voxel i. The following steps are then performed to generate a 3D heatmap of importance:

i. For each point (voxel center) in the MRI volume a vector $[x, y, z, r, g, b]$ is assigned, where x, y, z refer to the position of the point and r, g, b refer to the color of the point which shows its impact (or intensity) according to a pre-defined color-coding scheme (color map).
ii. The generated point cloud is then normalized (each x, y, z are normalized between 0 and 1)
iii. A desired surface within the MRI volume is specified. Since the impact of all voxels within the MRI volume is estimated and color-coded, it is possible to explore and visualize any desired areas throughout the MRI (or the volumetric ROI), for a comprehensive

understanding of how different intra- and peri-lesional regions contribute to the network's decisions in therapy outcome prediction.

iv. Since the number of points in the point cloud is limited, in order to improve the quality of the final 3D heatmap, interpolation is performed to generate new random points with the constraint of being on the specified surface.

v. Using a k-nearest neighbor algorithm the 10 closest points to each newly generated point are identified and their average color code is assigned it.

vi. The interpolated point cloud is used to calculate and generate surface normal orientations at each point required for surface reconstruction. Calculating normal orientations was done using a minimum spanning tree.

vii. Once the normal orientations are calculated, using the Poisson surface reconstruction techniques a smooth surface is generated showing important regions contributing to network decisions.

Figure S1 shows the overall procedure for generating a desired 3D surface from the initial point cloud.

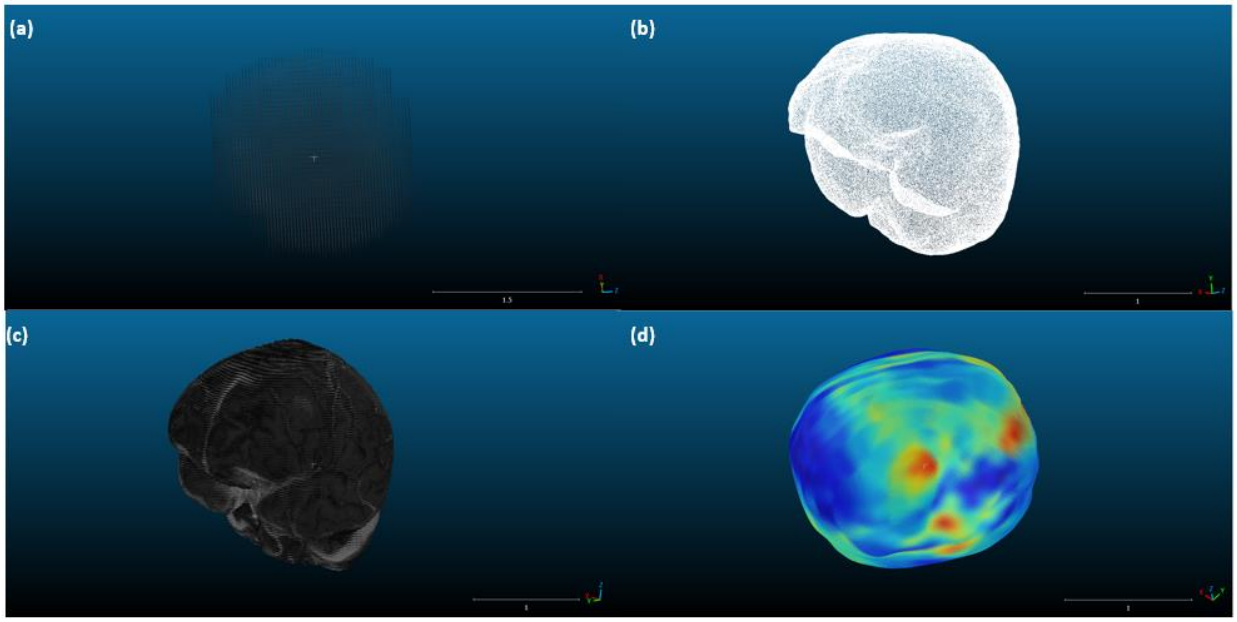

Figure S1 – The procedure for creating explorable 3D brain models and visualization heatmaps from a set of individual slices. **(a)** initially, the coordinates (x, y, z) of each voxel center in the MRI volume is determined. Since the number of slices is often limited, the resulting point cloud consists of multiple clusters with the same z and different x and y which is visually undesirable. To mitigate this issue, the points between slices are randomly interpolated, **(b)** the point cloud after the inter-slice interpolation, **(c)** the 3D brain model after assigning an intensity to each point in the point cloud and surface reconstruction, **(d)** applying the same procedure to generate a color-coded 3D visualization heatmap of importance for a lesion within the brain.

Supplementary Tables

Table S1. Patient Characteristics

Clinical Features / Outcome	Training Set (99 Patients and 116 lesions)	Test Set (25 patients and 40 lesions)
**Age**	62 ± 15 years	63 ± 17 years
**Gender**		
Male	39 patients (39%)	11 patients (44%)
Female	60 patients (61%)	14 patients (56%)
**Number of Brain Metastases**		
One lesion	34 patients (34%)	9 patients (36%)
Two lesions	35 patients (35%)	7 patients (28%)
Three lesions	11 patients (11%)	4 patients (16%)
More than three lesions	19 patients (19%)	5 patients (20%)
**Tumour Size (Longest Diameter)**	Range: 0.4 – 7 cm<br>Mean: 1.99 cm	Range: 0.6 – 6.6 cm<br>Mean: 2.06 cm
**Tumour Location**		
Supratentorium	87 lesions (75%)	29 lesions (72.5%)
Infratentorium	29 lesions (25%)	11 lesions (27.5%)
**Histology**		
Lung cancer	58 lesions (50%)	23 lesions (57.5%)
Breast cancer	26 lesions (22%)	9 lesions (22.5%)
Melanoma cancer	9 lesions (8%)	3 lesions (7.5%)
Colorectal cancer	7 lesions (6%)	0 lesions (0%)
RCC cancer	8 lesions (7%)	1 lesion (2.5%)
Other	8 lesions (7%)	4 lesions (10%)
**Total Dose (Over 5 Fractions)**		
22.5 Gy	1 lesion (1%)	0 lesions (0%)
25 Gy	20 lesions (17%)	8 lesions (20%)
27.5 Gy	6 lesions (5%)	2 lesions (5%)
30 Gy	73 lesions (63%)	20 lesions (50%)
32.5 Gy	7 lesions (6%)	6 lesions (15%)
35 Gy	9 lesions (8%)	4 lesions (10%)
**Previous WBRT**		
Yes	45 lesions (39%)	9 lesions (22.5%)
No	71 lesions (61%)	31 lesions (77.5%)
**Prior SRT/SRS**		
Yes	1 lesion (1%)	0 lesions (0%)
No	115 lesions (99%)	40 lesions (100%)
**Graded Prognostic Assessment (GPA)**		
0.00 –1.00	15 patients (15%)	3 patients (12%)
1.01–2.00	39 patients (39%)	14 patients (56%)
2.01–3.00	36 patients (36%)	3 patients (12%)
3.01– 4.00	9 patients (9%)	5 patients (20%)
**SRT Outcome**		
Crude LC	70 lesions (60%)	23 lesions (57.5%)
Crude LF	46 lesions (40%)	17 lesions (42.5%)

Table S2. Experimental results of the proposed framework on the validation set for 3D residual network, 3D residual network + CBAM attention, and 3D residual network + self-attention with different hyperparameters and pre-training settings.

Model	Pre-train (UCF101)	Pretrain (BraTS)	Learning Rate	Batch Size	Epochs	Accuracy	Sensitivity	Specificity
**3D Residual Network**	✓	✓	0.003	4	300	80%	66.7%	88.9%
**3D Residual Network + BiT**	✓	✓	0.003	4	300	80%	83.3%	77.8%
**3D Residual Network + CBAM Attention**	✓	✓	0.003	4	300	80%	83.3%	77.8%
**3D Residual Network + CBAM Attention + BiT**	✓	✓	0.003	4	300	80%	100%	66.7%
**3D Residual Network + Self-attention**	✓	✓	0.003	4	300	86.7%	83.3%	88.9%
**3D Residual Network + Self-attention + BiT**	✓	✓	**0.003**	**4**	**300**	**86.7%**	**83.3%**	**88.9%**
**3D Residual Network**	×	×	0.003	4	300	60%	66.7%	55.6%
**3D Residual Network**	×	✓	0.003	4	300	73.3%	66.7%	77.8%
**3D Residual Network + BiT**	×	✓	0.003	4	300	73.3%	83.3%	66.7%
**3D Residual Network + Self-attention + BiT**	×	✓	0.003	4	300	80%	83.3%	77.8%
**3D Residual Network + Self-attention + BiT**	✓	✓	0.00001	4	300	73.3%	83.3%	66.7%
**3D Residual Network + Self-attention + BiT**	✓	✓	0.01	4	300	80%	83.3%	77.8%
**3D Residual Network + Self-attention + BiT**	✓	✓	0.003	8	300	86.7%	100%	0.78%

Table S3. Table 1. Results of radiotherapy outcome prediction for different models on the training, validation, and test sets. Acc: Accuracy; Sens: sensitivity; Spec: specificity.

Network	Train Set					Validation Set					Independent Test Set				
	**Acc.**	**Sens.**	**Spec.**	**AUC**	**F1-Score**	**Acc.**	**Sens.**	**Spec.**	**AUC**	**F1-Score**	**Acc.**	**Sens.**	**Spec.**	**AUC**	**F1-Score**
**3D Residual Network**	85%	75%	91.8%	0.88	80%	80%	66.7%	88.9%	0.84	72.7%	80%	71%	**87%**	0.83	75%
**3D Residual Network + BiT**	85%	77.5%	90%	0.89	80%	80%	83.3%	77.8%	0.86	76.9%	80%	82.4%	78.%	0.84	77.8%
**3D Residual Network + CBAM Attention**	87%	80%	91.8%	0.95	83.1%	80%	83.3%	77.8%	0.88	76.9%	80%	82.4%	78.2%	0.87	77.8%
**3D Residual Network + CBAM Attention + BiT**	87%	**82.5%**	90%	0.95	83.5%	80%	100%	66.7%	0.88	80%	80%	**88.2%**	73.9%	0.88	78.9%
**3D Residual Network + Self-attention**	89%	**82.5%**	93.5%	0.97	85.7%	**86.7%**	**83.3%**	**88.9%**	0.89	**83.3%**	**82.5%**	76.5%	**87%**	0.88	78.8%
**3D Residual Network + Self-attention + BiT**	**90%**	**82.5%**	**95%**	**0.98**	**86.8%**	**86.7%**	**83.3%**	**88.9%**	**0.93**	**83.3%**	**82.5%**	82.4%	82.6%	**0.91**	**80%**
